# Valorization of the Antioxidant Effect of Mantua PGI Pear By-Product Extracts: Preparation, Analysis and Biological Investigation

**DOI:** 10.3390/antiox12010144

**Published:** 2023-01-07

**Authors:** Carlotta Bollati, Stefania Marzorati, Lorenzo Cecchi, Martina Bartolomei, Jianqiang Li, Maria Bellumori, Lorenza d’Adduzio, Luisella Verotta, Laura Piazza, Anna Arnoldi, Nadia Mulinacci, Carmen Lammi

**Affiliations:** 1 Department of Pharmaceutical Sciences, University of Milan, 20133 Milan, Italy; 2 Department of Environmental Science and Policy, Università degli Studi di Milano, 20133 Milan, Italy; 3 Pharmaceutical and Nutraceutical Section, Department of Neuroscience, Psychology, Drug and Child Health, University of Florence, 50019 Florence, Italy

**Keywords:** antioxidant activity, circular economy, Mantua pear, pear extracts, supercritical CO_2_ extraction

## Abstract

For improving the management of the production chain of PGI Mantua pears (which comprises many varieties, including Abate Fetel), applying the cardinal principles of circular economy and sustainability, the fruits with diseases or defects were recovered for producing dried rounds of pears from the Abate Fetel cultivar, a new product with high nutritional value that extends the remaining life. This process led to the production of secondary and residual by-products, which are mainly composed of the highest and lowest part of the fruits, comprising seeds, pulps, peels and petioles. Hence, this study was focused on the valorization of these secondary by-products of Abate Fetel pears through the production of pear extracts using traditional and “green” extraction methods that involve the use of supercritical CO_2_ fluid extraction. The produced extracts, together with a reference solvent-derived extract, were analyzed by HPLC-ESI-MS, and in parallel, their direct and cellular antioxidant activity were assessed. Evidence has indicated that all the tested extracts reduced the H_2_O_2_-induced reactive oxygen species (ROS), lipid peroxidation and nitric oxide (NO) levels, respectively, in human intestinal Caco-2 cells. Hence, this study clearly suggests that extracts obtained from Mantuan PGI pear by-products may be used as valuable sources of bioactive upcycled phytocomplex for the development of dietary supplements and/or functional foods.

## 1. Introduction

Many chronic diseases, such as autoimmune disorders, cancer and metabolic and cardiovascular diseases, are positively correlated with the imbalance of antioxidants and prooxidants in favor of prooxidants, leading to the formation of high levels of reactive oxygen species (ROS) [1]. Indeed, oxidative stress is one of the main risk factors for the generation of multifactorial pathological conditions; however, several epidemiological studies have provided evidence that consuming foods that are rich in antioxidants may reduce this risk [2]. Notably, fruits and vegetables are an excellent source of substances with antioxidant and pro-health properties, mainly polyphenols, carotenoids and triterpenoids [3]. In this context, preliminary investigations have suggested that pear fruits (*Pyrus communis* L.) are a valuable source of bioactive compounds with health-promoting effects [3,4].

In Italy, the area of Mantua in the Lombardy region—in particular, the Oltrepò area—boasts one of the most interesting and ancient traditions of quality pear cultivation. Indeed, Pera Mantovana has ancient origins dating back to at least the fourteenth century. Its primacy as the most important cultivation in the production area was already reported in 1475 (https://www.qualigeo.eu/en/product/pera-mantovana-pgi/, accessed on 2 December 2022). However, it was only in the mid-twentieth century that the production reached a commercial level, as this fruit was used almost exclusively for local consumption until the end of the Second World War. In more recent times, pear cultivation has developed considerably, above all thanks to technological innovations in the preserving and transport sectors (https://www.qualigeo.eu/en/product/pera-mantovana-pgi/, accessed on 2 December 2022).

According to the regulations acknowledging its Protected Geographical Indication (PGI), Pera Mantovana is produced in 27 municipalities of the province and comprises the following pear varieties: William, Max Red Bartlett, Conference, Decana del Comizio, Abate Fetel and Kaiser. Abate Fetel pears are characterized by a thin, yellowish and slightly “rusty” skin around the stalk, sometimes suffused with red on the side most exposed to the sun, and have a white, juicy pulp with a sweet–sour taste.

For improving the management of the production chain of PGI Mantua pears by applying the cardinal principles of circular economy and sustainability, inside a collaborative project, Abate Fetel fruits with diseases or defects were recovered for producing dried rounds of pear—a new product with a high nutritional value that extends the remaining life. This process led to the production of secondary and residual by-products, which are mainly composed of the highest and lowest parts of the fruits, comprising seeds, pulps, peels and petioles. Hence, this study was focused on the valorization of these secondary by-products of Abate Fetel pears through the production of pear extracts using traditional and “green” extraction methods. More in detail, a “green” extraction was developed by means of supercritical carbon dioxide. This technology, employing CO_2_ at its supercritical conditions with a small percentage of ethanol as the cosolvent, is indeed characterized by low environmental impacts, because the use of organic solvents is minimized, ensuring safe and selective processes directly on the biomass without any pretreatment, with the possibility to recycle the employed CO_2_ in industrial plants. In parallel, a traditional solvent-based extraction was carried out as a reference condition employing ethyl acetate. Afterwards, selected extracts were analyzed by HPLC-DAD-MS in order to identify their chemical composition. In parallel, the biological properties were assessed combining in vitro and cellular techniques. Firstly, the direct antioxidant activity of each pear extract was investigated using the 2,2-diphenyl-1-picrylhydrazyl (DPPH), 2,2-azino-bis-(3-ethylbenzothiazoline-6-sulfonic acid (ABTS) and ferric reducing antioxidant power (FRAP) assays. Secondly, the effects of pear extracts on the human intestinal Caco-2 cell vitality was performed by 3-(4,5-dimethylthiazol-2-yl)-2,5-diphenyltetrazolium bromide (MTT) experiments. Finally, Caco-2 cells were treated with H_2_O_2_ in order to induce oxidative stress, and then, their ability to modulate the H_2_O_2_-induced ROS, lipid peroxidation and nitric oxide (NO) and inducible nitric oxide synthase (iNOS) pathways were verified after pretreatment with each pear extract (2 mg/mL).

## 2. Materials and Methods

### 2.1. Chemicals

Dulbecco’s modified Eagle’s medium (DMEM), L-glutamine, fetal bovine serum (FBS), phosphate-buffered saline (PBS), penicillin/streptomycin, chemiluminescent reagent and 24- or 96-well plates were purchased from Euroclone (Milan, Italy). MTT (3-(4,5-dimethylthiazol-2-yl)-2,5-diphenyltetrazolium bromide), DPPH (1,1-diphenyl-2-picrylhydrazyl), 2,2-azino-bis-(3-ethylbenzothiazoline-6-sulfonic acid (ABTS), TPTZ, Griess reagent, bovine serum albumin (BSA), RIPA buffer, the antibody against β-actin, the fluorometric intracellular ROS kit and MDA assay kit were bought from Sigma-Aldrich (St. Louis, MO, USA). Phenylmethanesulfonylfluoride (PMSF), Na-orthovanadate inhibitors and the antibodies against rabbit Ig-horseradish peroxidase (HRP) and mouse Ig-HRP were purchased from Santa Cruz Biotechnology Inc. (Santa Cruz, CA, USA). The iNOS primary antibody came from Cell Signaling Technology (Danvers, MA, USA); the inhibitor cocktail Complete Midi from Roche (Basel, Swiss); mini-protean TGX pre-cast gel 7.5% and mini-nitrocellulose Transfer Packs from Bio-Rad (Hercules, CA, USA). Ultrapure water was produced using a Milli-Q System (Millipore SA, Molsheim, France). Acetonitrile with a HPLC grade was from Panreac (Barcelona, Spain); formic acid was from Merck (Darmstadt, Germany). Standards of quercitrin (>98.5%) and ursolic acid (>98.0%) were from Extrasynthese (Genay, France).

### 2.2. Pear Extract Preparation

#### 2.2.1. Starting Biomass

The pear waste biomass (*Pyrus communis* L. cultivar ‘Abate Fétel’, 3 kg) was provided by CREA (Consiglio per la Ricerca in Agricoltura e l’Analisi dell’Economia Agraria) after a sliding process to produce dried pear disks to be consumed as food. Prior to the drying process, the upper and lower parts of each fresh pear, containing the skin and the core pulp with seeds and stalks, characterized by a noncompliant disk diameter, were discarded as a waste and stored at −20 °C in inert atmosphere. Pear waste was chopped and then lyophilized for 24 h using a Cinquepascal S.R.L. (Trezzano Sul Naviglio, Italy) freeze dryer equipment. The lyophilized material was then ground using a knife mill (Fritsch, Pulverisette 11, Fritsch GmbH—Milling and Sizing, Idar-Oberstein, Germany) at 10,000 rpm for 20 s. To avoid powder heating during blending and the consequent degradation of thermally unstable species, liquid nitrogen was added. The pulverized biomass was then stored at −20 °C.

#### 2.2.2. Extractions

##### Solvent-Based Extraction

The lyophilized and pulverized biomass (19.4 g) was transferred in a 500 mL round flask and extracted with 150 mL of ethyl acetate, heated up to the boiling point with continuous stirring with a magnetic stirrer at reflux for 2.5 h. After cooling to room temperature, the suspension was filtered by means of a Buchner funnel to remove the solid particles. The extract (EtOAc) was then dried by a rotary evaporator (37 °C) and, finally, by a mechanical vacuum pump. The extraction was run in triplicate.

##### Supercritical Fluid Extraction

Supercritical fluid extractions were performed using a pilot unit SFT110XW System supplied by Supercritical Fluid Technologies Inc. (Newark, DE, USA). It consisted of a 100 cm^3^ stainless steel extractor inserted in an oven, a constant pressure piston pump (SFT-Nex10 SCF Pump) with a Peltier cooler, a Waters 515 HPLC pump (Waters, Milfors, MA, US) for the cosolvent and a collection vial. The lyophilized and pulverized biomass (40.7 g) was loaded in the vessel for supercritical fluid extraction. The restrictor temperature was set at 75 °C. Sequentially different conditions (changing the pressure (p), temperature (T) and/or the amount of cosolvent) were applied, each one comprising an alternation of 15 min in static conditions (maceration in supercritical CO_2_) followed by 30 min in dynamic conditions (flow rate CO_2_ = 8.0 SCFH, standard cubic feet per hour):

- p = 150 bar, T = 40 °C (d_CO2_ = 780.6 kg/m^3^). The alternation of 3 static/dynamic cycles was enough to exhaust the extractables in these conditions, and no evident mass gain was further achieved (SF1).
- p = 300 bar, T = 40 °C (d_CO2_ = 909.3 kg/m^3^). These conditions were not able to provide any extract mass gain.
- p = 300 bar, T= 60 °C (d_CO2_ = 829.5 kg/m^3^). These conditions were not able to provide any extract mass gain.
- p = 300 bar, T= 80 °C (d_CO2_ = 746.1 kg/m^3^). The alternation of 1 static/dynamic cycle was enough to exhaust the extractables in these conditions, and no evident mass gain was further achieved.
- p = 300 bar, T= 60 °C, cosolvent = ethanol (10% v _EtOH_/v _sc-CO2_). An alternation of 7 static/dynamic cycles was carried out (SF6).

Samples were dried by a rotary evaporator (37 °C) and, finally, by a mechanical vacuum pump. Only the extracts deriving from the first (SFE1) and second-last (SFE6) cycles in the presence of ethanol were considered promising samples and were thus further investigated.

#### 2.2.3. Waxes Removal

Before analysis, the three samples EtOAc, SFE1 and SFE6 were subjected to a procedure aimed at removing the waxes and explained in the Supplementary Materials.

### 2.3. HPLC-DAD-MS Analysis of Pear Extracts

The dried extracts of the three samples were dissolved in ethanol to obtain a final concentration of 10 mg/mL each. The analyses were carried out using the 1260 Infinity II LC System coupled with both a Diode Array Detector and a Mass Spectrometry Detector (InfinityLab LC/MSD, Agilent Technologies, CA, USA) with an API electrospray interface (all from Agilent Corporation, Santa Clara, CA, USA). The column was a Poroshell 120, EC-C18 (150 mm × 3.0 mm id, 2.7 µm, Agilent Technologies, CA, USA). The mobile phase was constituted by acetonitrile (A) and water at pH 3.2 by formic acid (B); the flow rate was 0.4 mL min^−1^. A multistep linear gradient was applied: it started with A 5% at 0 min, from 5% to 40% in 40 min, a plateau at 40% until 45 min, then A 70% at 50 min, a plateau until 60 min and, finally, to A 100% at 65 min, and the last step was a plateau until 68 min; the system returned at A 5% in 2 min. The chromatograms were recorded at wavelengths set at 210, 240, 280 and 350 nm.

The mass spectra were acquired in the negative ion mode applying the following conditions: range mass acquisition 100–1000 Dalton, gas temperature 350 °C, nitrogen flow rate 12 L/min, nebulizer pressure 35 psi, capillary voltage 3500 V and fragmentation energy between 100 and 200 V.

The total content of flavonoids was evaluated at 350 nm by the calibration line of quercitrin at 350 nm (linearity range 0–0.49 µg, R^2^ = 0.9999). Ursolic acid (a triterpenoid compound) was determined using a calibration curve at 220 nm (linearity range 0–12 µg; R^2^ = 0.9999) of the pure standard.

### 2.4. DPPH (2,2-Diphenyl-1-picrylhydrazyl Radical Scavenging) Assay

The 1,1-Diphenyl-2-picrylhydrazyl radical (DPPH) assay was performed to determine the antioxidant activity by the standard method [5] with a slight modification. The major details are reported in the Supplementary Materials.

### 2.5. TEAC Assay

The TEAC assay is based on a reduction of the 2,2-azino-bis-(3-ethylbenzothiazoline-6-sulfonic acid (ABTS) radical induced by antioxidants [6]. The ABTS radical cation ABTS^+●^ was prepared by mixing a 7 mM ABTS solution (Sigma-Aldrich, Milan, Italy) with 2.45 mM potassium persulfate (1:1) and stored for 16 h at room temperature and in the dark. The preparation of the ABTS reagent and the assay procedure described in the Supplementary Materials.

### 2.6. FRAP Assay

The FRAP assay evaluates the capacity of a compound to reduce ferric ion (Fe^3+^) into ferrous ion (Fe^2+^) [7]. The assay procedure is reported in the Supplementary Materials.

### 2.7. Cell Culture

Caco-2 cells, obtained from INSERM (Paris, France), were routinely subcultured at 50% density and maintained at 37 °C in a 90% air/10% CO_2_ atmosphere, following a procedure previously reported [8] and described more in the Supplementary Materials.

### 2.8. 3-(4,5-Dimethylthiazol-2-yl)-2,5-Diphenyltetrazolium Bromide (MTT) Assay

The MTT experiments were conducted on Caco-2 cells following the procedure previously reported [9] and completely described in the Supplementary Materials.

### 2.9. Nitric Oxide Level Evaluation on Caco-2 Cells

Caco-2 cells (1.5 × 10^5^/well) were seeded on 24-well plates. The next day, cells were treated for 24 h with EtOAc, SFE1 or SFE6 to reach the final concentration of 2.0 mg/mL and incubated at 37 °C under a 5% CO_2_ atmosphere. After incubation, cells were treated with H_2_O_2_ (1.0 mM) or a vehicle for 1 h, and then, the cell culture media were collected for the experiment evaluations. More details are reported in the Supplementary Materials.

### 2.10. Fluorometric Intracellular ROS Assay

For cell preparation, 3 × 10^4^ Caco-2 cells/well were seeded on a black 96-well plate in growth medium. After 24 h, the medium was removed and replaced with 50 μL/well of the Master Reaction Mix for 1 h in the dark. Then, the cells were treated following a method reported in the Supplementary Materials.

### 2.11. Lipid Peroxidation (MDA) Assay

Caco-2 cells (2.5 × 10^5^ cells/well) were seeded in a 24-well plate, and the following day, they were treated with 2.0 mg/mL of EtOAc, SFE1 or SFE6 for 2 h at 37 °C under 5% CO_2_ atmosphere. After incubation, the cells were stimulated with 1 mM H_2_O_2_ or a vehicle for 30 min, then collected for the experiments. The detailed method description is reported in the Supplementary Materials.

### 2.12. Western Blot Analysis

1.5 × 10^5^ Caco-2 cells/well (24-well plate) were treated with 2 mg/mL of EtOAc, SFE1 and SFE6 for 24 h. After incubation, the cells were stimulated with H_2_O_2_ (1.0 mM) or a vehicle for 1 h, and then, the cell culture media were collected in an ice-cold microcentrifuge tube and processed for the Griess assay. Meanwhile, the cells were scraped in 30 µL ice-cold lysis buffer (RIPA buffer + inhibitor cocktail + 1:100 PMSF + 1:100 Na-orthovanadate), transferred in an ice-cold microcentrifuge tube and centrifuged for the proteins’ isolation. The procedure is explored more in the Supplementary Materials.

### 2.13. Statical Analysis

All results were expressed as the mean ± standard deviation (s.d.), where *p*-values < 0.05 were considered to be significant. Statistical analyses were performed by one- and two-way ANOVA followed by Tukey’s post-test, respectively (GraphPad Prism 9, GraphPad Software, La Jolla, CA, USA).

## 3. Results

### 3.1. Preparation of Pear Extract Exploiting Traditional and Green Extraction Methods

In the experiment setup, a conventional solvent-based extraction with ethyl acetate was at first performed, modifying some literature protocols [10,11], in order to set a reference when comparing results from supercritical fluid extractions. By means of this methodology, an extraction yield of 1.0 ± 0.1% was obtained.

When supercritical CO_2_ was employed as an extraction strategy, the following results were achieved:

- p = 150 bar, T = 40 °C. An overall extraction yield of 0.10 ± 0.02% was achieved.
- p = 300 bar, T = 40 °C. These conditions were not able to provide any extract mass gain.
- p = 300 bar, T = 60 °C. These conditions were not able to provide any extract mass gain.
- p = 300 bar, T = 80 °C. The extraction yield was slightly incremented to 0.11% (0.01% yield gain).
- p = 300 bar, T = 60°, cosolvent = ethanol (10% v _EtOH_/v _sc-CO2_). After the alternation of seven static/dynamic cycles, the final yield was incremented up to 1.1 ± 0.1% (1.0% yield gain).

**Figure 1:**
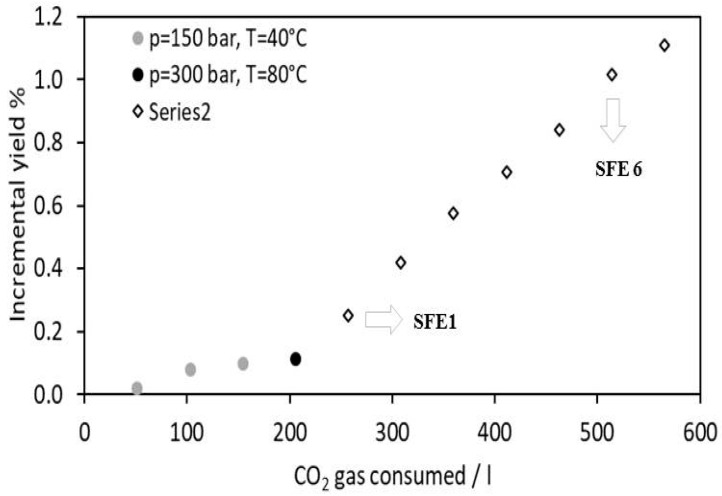
Supercritical CO_2_ extraction kinetics: each point represents the incremental yield (% mass extract/mass biomass) detected over the time and, hence, over CO_2_ consumption.

Figure 1 displays the incremental yields obtained over the time and, hence, over CO_2_ consumption in all the tested extraction conditions.

Samples of EtOAc, SFE1 and SFE6 selected for further investigations were dewaxed in order to decrease the presence of apolar compounds. The waxes content removed from the EtOAc, SFE1 and SFE6 samples was 24, 10 and 7.5% by weight, respectively.

### 3.2. Analysis of the Pear Extracts

**Figure 2:**
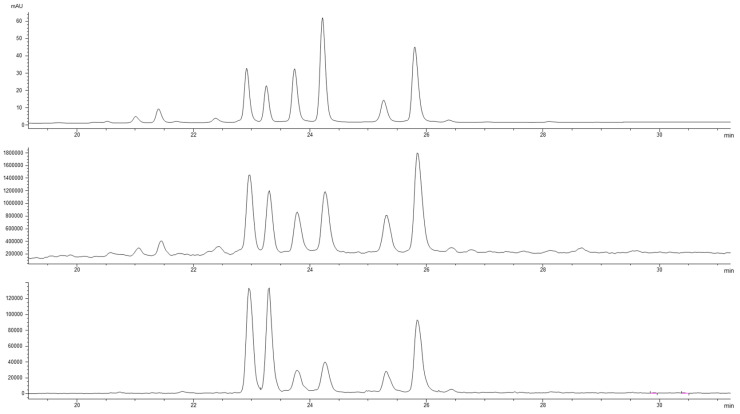
Chromatographic profiles at 350 nm of the SFE1 sample with the corresponding total ion current in the negative ionization mode and the EI at 315 *m*/*z* corresponding to the aglycone for the isorhamnetin derivatives; (**1**,**2**) quercetin 3-O glucosides; (**3**,**4**) isorhamnetin 3-O glucorhamnosides; (**5**,**6**) isorhamnetin 3-O glucosides; (**7**,**8**) isorhamnetin acetyl hexosides.

The literature [10] indicates that three main terpenoids are generally detected in pear samples, with ursolic acid as the largely prevalent molecule. The presence of ursolic acid in our samples was confirmed by HPLC-DAD using the pure standard evaluated at 220 nm, whereas the other components of the extracts were identified by HPLC-ESI-MS. The chromatographic profiles at 350 nm, the corresponding profiles in negative ionization mode and the extract ion at 315 *m*/*z* (Figure 2) allowed the identification of the main flavonols (Table S1). In accordance with the literature data [10,12,13], it was possible to detect two minor isobars of quercetin hexoside (**1,2**) and a second group that included three isobaric pairs of isorhamnetin glycosides with two monohexosides (**3**,**4**), two di-glycosides (**5**,**6**) and two acyl esters of the monoexosides (**7**,**8**). The very similar UV–Vis spectra of the eight flavonols (Figure S1) suggested the glycosidic moieties were all linked in the same position of the aglycone. The mass spectra allowed distinguishing the two groups of flavonoids by the presence of the respective aglycone ions at 301 *m*/*z* for the quercetin derivatives and 315 *m*/*z* for the isorhamnetin ones. In accordance with the data bank of phenol-explorer for the pear fruit, it was hypothesized that all the mono-hexosides were linked to the hydroxyl group in three of the C rings of the flavonols (http://phenol-explorer.eu/contents/polyphenol/326, accessed on 2 December 2022). Consequently, the mono-glycosides of quercetin (**1**,**2**) and of isorhamnetin (**5,6**) were recognized as 3-O glucosides or 3-O galactosides. As for the pair of isorhamnetin glucorhamnosides (**3**,**4**), their mass spectra showed the absence of ions correlated to the presence of monoglucoside or monorhamnoside species, and the loss of 308 Dalton indicated the presence of a disaccharide group in the molecule. They were tentatively identified as 3-O glucorhamnoside derivatives. The last pair of flavonols, two acetyl-glycosylated forms of isorhamnetin (**7,8**), showed the highest retention times in accordance with the presence of the ester group in the molecule; the loss of 204 Dalton from the molecular ion in the mass spectra confirmed the presence of the acetyl-glycoside moiety in the two molecules. The list of the identified compounds in Table S1 was in accordance with the previous data on the phenolic compounds in pears [10,13].

**Figure 3:**
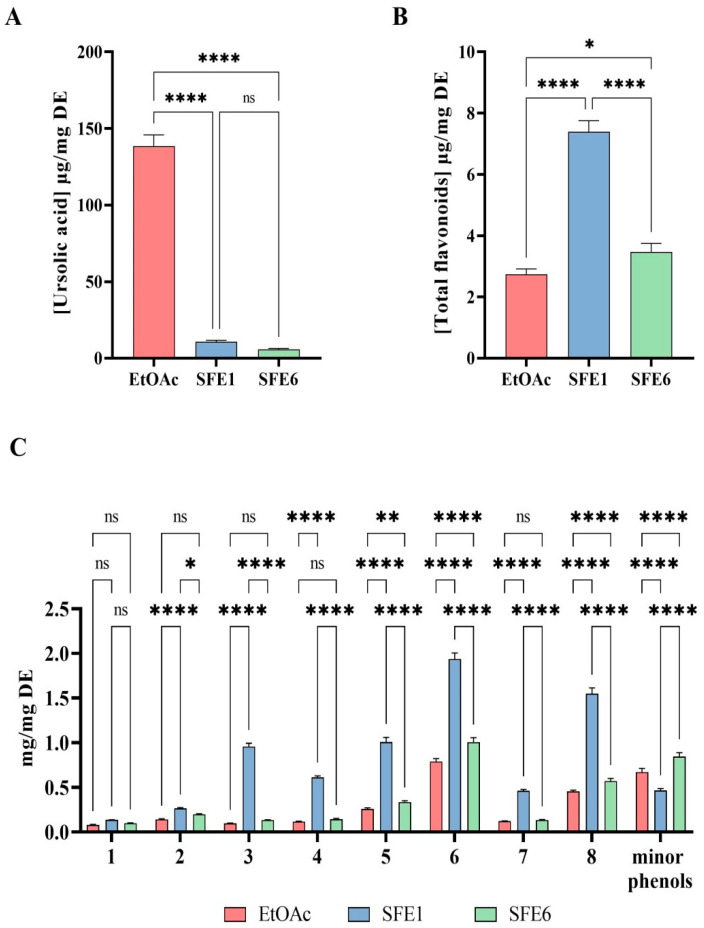
Total amount of ursolic acid (**A**), sum of flavonols and minor phenols (**B**) and distribution of each flavonols (**C**) evaluated on dried extract (DE). The data are a mean of three independent determinations. Data sets were analyzed by one-way ANOVA and two-way ANOVA followed by Tukey’s post-hoc test. Ns: not significant; C: control sample; (*) *p* < 0.05, (**) *p* < 0.01 and (****) *p* < 0.0001.

A comparison of the EtOAc and SFE6 samples showed that their profiles at 350 nm were almost overlapped, while the SFE1 sample was characterized by a higher concentration of all the flavonoids, as shown in Figure 3B,C.

As for the ursolic acid concentration, the traditional extraction provides an efficient recovery of this triterpenoid (Figure 3A), reaching a concentration on dry extract up to 15 times higher than that observed using the supercritical extraction. Therefore, it is possible to affirm that the supercritical fluid extraction, although modified with a small percentage of ethanol, was not efficient in extracting the ursolic acid.

The concentration of the flavonoids in the dried extracts was evaluated using the quercetin 3-*O*-glucoside at 350 nm as an external standard. The results clearly showed that the supercritical extraction increases the total amount of flavonoids. In particular, the SFE1 extract was the richest sample, followed by SFE6. Differently from the results of ursolic acid, in this case, the extraction by the supercritical fluid gave the best quantitative results, particularly for the SFE1 sample.

### 3.3. Evaluation of the Direct Antioxidant Activity of EtOAc, SFE1 and SFE6

**Figure 4:**
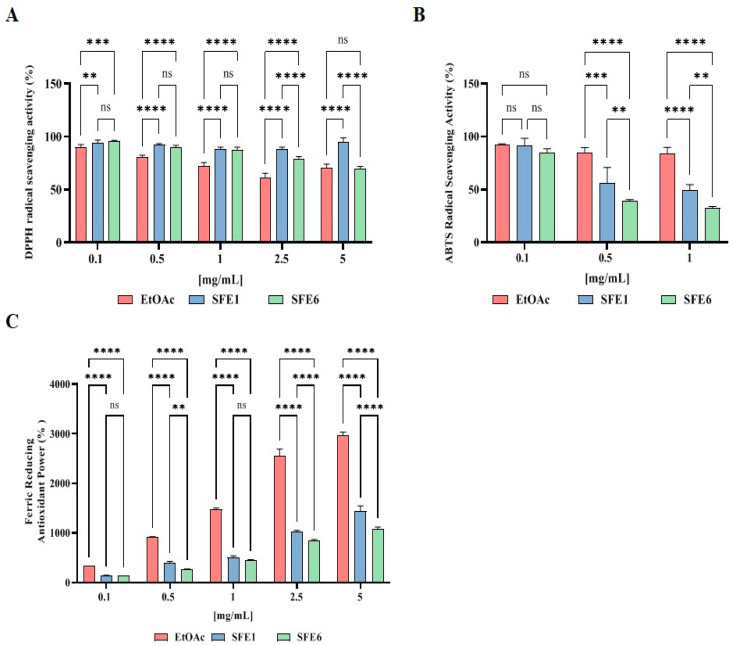
(**A**) In vitro radical scavenging activity by the DPPH assay. (**B**) In vitro radical scavenging activity by the ABTS assay. (**C**) In vitro radical scavenging activity by the FRAP assay. Data represent the mean ± s.d. of three determinations performed in triplicate. All the data sets have been analyzed by two-way ANOVA followed by Šidák correction. ns: not significant, (**) *p* < 0.01, (***) *p* < 0.001 and (****) *p* < 0.0001.

In order to assess the direct antioxidant properties of the extracts, the DPPH, ABST and FRAP assays were performed (Figure 4). As reported in Figure 4A, at the concentration of 0.1 mg/mL, EtOAc, SFE1 and SFE6 scavenged the DPPH radical by 9.8 ± 2.3%, 5.7 ± 2.4% and 4.3 ± 1.0%, respectively; at the concentration of 0.5 mg/mL by 19.0 ± 1.5%, 7.8 ± 1.2% and 10.2 ± 1.9%, respectively; at the concentration of 1 mg/mL, by 27.6 ± 3.1%, 11.4 ± 1.4% and 12.9 ± 2.8%, respectively. Tested at 2.5 and 5 mg/ml, they scavenged the DPPH radical by 38.4 ± 3.8% and 29.7 ± 3.8%, 11.8 ± 1.9% and 5.3 ± 4.2% and 21.2 ± 2.2% and 30.4 ± 1.9%, respectively (Figure 4A).

The results indicate that EtOAc, SFE1 and SFE6 scavenged the ABTS radical by 7.2 ± 0.5%, 8.7 ± 7.2% and 14.8 ± 3.4 at 0.1 mg/mL respectively; they scavenged the ABTS radical by 15.1 ± 4.6%, 43.7 ± 14.5% and 60.9 ± 1.4% at 0.5 mg/mL, respectively. Finally, tested at 1 mg/mL, the ABTS radical was reduced by 15.9 ± 5.7%, 50.8 ± 5.5% and 67.5 ± 1.4% for EtOAc, SFE1 and SFE6, respectively (Figure 4B).

In Figure 4C, the results show that EtOAc, SFE1 and SFE6 increased the FRAP by 330.1 ± 2.1%, 145.7 ± 3.2% and 133.0 ± 1.8% at 0.1 mg/mL, respectively, and they improved the FRAP by radical by 907.5 ± 13.4%, 387.2 ± 36.1% and 272.1 ± 4.1 at 0.5 mg/mL, respectively. For the 1 mg/mL treatments, the FRAP levels were increased by 1477.8 ± 19.6%, 502.8 ± 32.5% and 449.0 ± 11.42 for EtOAc, SFE1 and SFE6, respectively. Finally, EtOAc, SFE1 and SFE6 tested at 2.5 and 5 mg/mL improved the FRAP by 2558.0 ± 130.0% and 2963.6 ± 66.7%, 1020.2 ± 25.6% and 1432.5 ± 108.7% and 835.8 ± 28.1% and 1083.0 ± 34.2%, respectively (Figure 4C).

### 3.4. Evaluation of Caco-2 Cells Viability

On the basis of these positive results, it was decided to carry out the cellular evaluations of the antioxidant properties of EtOAc, SFE1 and SFE6. Before proceeding to in situ experiments on Caco-2 cells, it was necessary to perform the MTT experiments to verify that the samples did not exhibit cytotoxicity. The results suggested that all the samples were safe for intestinal cells at all the concentrations tested (Figure S1).

### 3.5. EtOAc, SFE1 and SFE6 Decrease the H_2_O_2_-Induced ROS and Lipid Peroxidation Levels in Human Intestinal Caco-2 Cells

**Figure 5:**
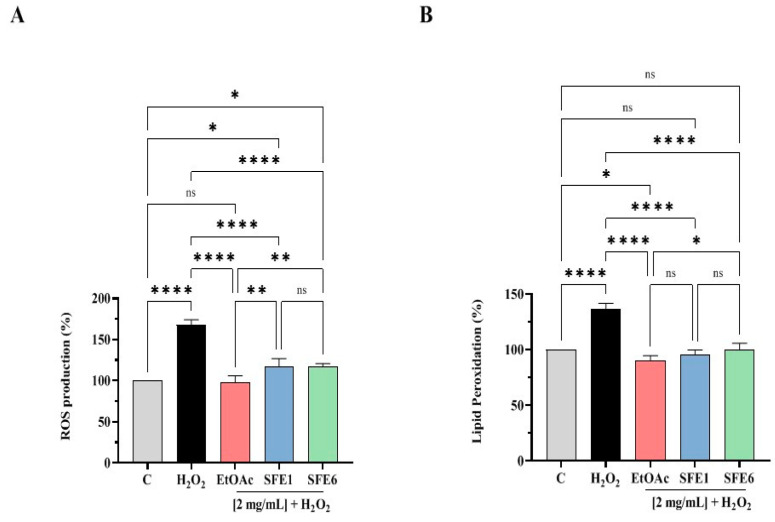
(**A**) Modulation of the intracellular ROS level H_2_O_2_-induced after the pretreatment with EtOAc, SFE1 and SFE6. (**B**) Modulation of H_2_O_2_-induced lipid peroxidation after the pretreatment with EtOAc, SFE1 and SFE6. Data represent the mean ± s.d. of six independent experiments performed in triplicate. All data sets were analyzed by one-way ANOVA followed by Tukey’s post-hoc test. C: control sample; ns: not significant; (*) *p* < 0.05, (**) *p* < 0.01 and (****) *p* < 0.0001.

To evaluate the ability of the samples to varied Reactive Oxygen Species (ROS) overproduction induced by H_2_O_2_, cellular experiments were conducted. Caco-2 cells treated with H_2_O_2_ alone showed an increase of ROS levels up to 167.6 ± 6.37% versus the control cells. On the contrary, the EtOAc, SFE1 and SFE6 samples (2 mg/mL) modulated the intracellular ROS levels by 97.48 ± 8.47%, 116.9 ± 10.02% and 116.8 ± 3.96%, respectively, after the induction of oxidative stress mediated by H_2_O_2_ (Figure 5A).

In addition, for evaluating the capacity of the samples to modulate the H_2_O_2_-induced lipid peroxidation in human intestinal Caco-2 cells, the MDA measurement was assessed. According to the observed increase of ROS after the H_2_O_2_ treatment, an increase of the lipid peroxidation was observed up to 136.7 ± 4.94% versus the control cells; however, the pretreatment with EtOAc, SFE1 and SFE6 (2 mg/mL) resulted in a decrease in the MDA levels up to 90.32 ± 4.36%, 95.33 ± 4.39% and 100.3 ± 5.40%, restoring the lipid peroxidation baseline levels (Figure 5B).

### 3.6. EtOAc, SFE1 and SFE6 Modulate the H_2_O_2_-Induced NO Level Production via the iNOS Protein Modulation in Caco-2 Cells

**Figure 6:**
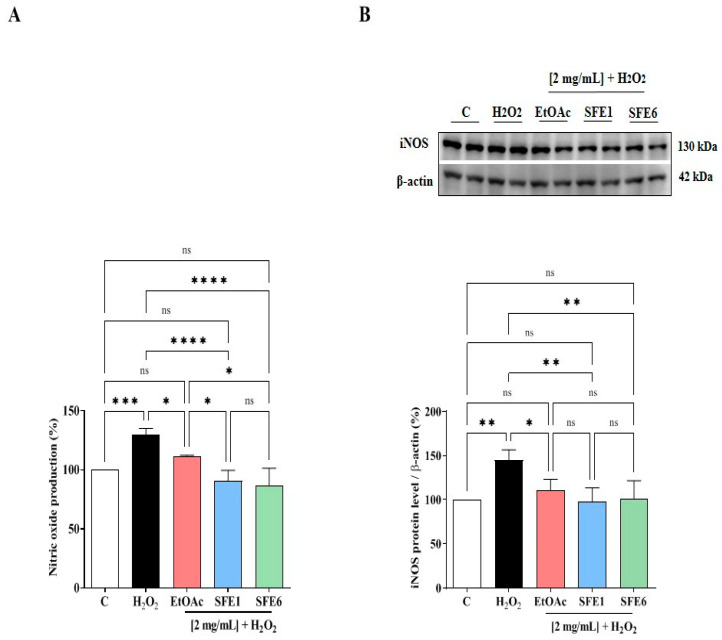
Effect of EtOAc, SFE1 and SFE6 on the H_2_O_2_ (1 mM)-induced NO production (**A**) and inducible nitric oxide synthase (iNOS) protein levels (**B**) in human intestinal Caco-2 cells. The data points represent the averages ± s.d. of four independent experiments in duplicate. All data sets were analyzed by one-way ANOVA followed by Tukey’s post-hoc test. C: control sample; ns: not significant; (*) *p* < 0.05, (**) *p* < 0.01, (***) *p* < 0.001 and (****) *p* < 0.0001.

The effects of EtOAc, SFE1 and SFE6 on NO production were evaluated on human intestinal Caco-2 cells after oxidative stress induction. The H_2_O_2_ (1 mM) treatment induced an oxidative stress that led to an increase of the intracellular NO levels up to 124.4 ± 4.32% (Figure 6A). The pretreatment with EtOAc, SFE1 and SFE6 (2 mg/mL) reduced the H_2_O_2_-induced NO overproduction up to 111.71 ± 0.70%, 90.76 ± 8.75% and 86.26 ± 15.11%, respectively, decreasing their values close to the basal levels (Figure 6A).

In parallel, the effects of EtOAc, SFE1 and SFE6 on the iNOS protein levels were assessed after oxidative stress induction by Western blot experiments, in which the iNOS protein band at 130 kDa was detected and quantified. Our results (Figure 6B) clearly demonstrated that, after H_2_O_2_ treatment (1 mM), the iNOS protein increased up to 144.7 ± 11.73% in Caco-2 cells. In agreement with the modulation of NO production, the pretreatment of Caco-2 cells with EtOAc, SFE1 and SFE6 reduced the H_2_O_2_-induced iNOS protein, bringing their levels close to the basal conditions. In particular, EtOAc reduced the iNOS levels up to 110.31 ± 12.8%, SFE1 reduced up to 97.33 ± 16.17% and SFE6 decreased up to 101.19 ± 20.45%, respectively, at 2 mg/mL (Figure 6B).

## 4. Discussion

Pears are one of the most widely consumed fruits [14], and they are an important crop in Italy, which is the most important area of production in Europe [15]. Some recent evidence suggests that pear extracts exert interesting biological activities [14], which may contribute to explain the reason why this fruit has been used as folk medicine and a healthy food for more than two millennia in some Asian countries (i.e., Korea and China) [14]. Indeed, in vitro, in vivo and human studies demonstrate that pears exert hypoglycemic, anti-obese, anti-inflammatory, antitumoral, hypolipidemic and skin whitening effects [14,16,17,18], even though the mechanisms of action at the bases of these effects are still poorly elucidated. Actually, most of the studies on the health-promoting activity of pears are focused on the peeled fruits, although most of the pear active components (e.g., flavonoids, triterpenoids and phenolic acids) are mainly present in the fruit peels.

In general, fruits with some defects are available at discount markets or are treated as “wastes”. Applying the cardinal principles of circular economy and with the aim at fostering and improving the sustainability of Mantuan PGI pears, in this work, we demonstrated that Italian Abate Fetel cultivar by-products may become an interesting source of active compounds with antioxidant activity.

More in detail, a conventional ethyl acetate extraction method was at first carried out in order to set a benchmark and compare the results obtained with more modern methodologies. A “medium polarity” solvent, such as ethyl acetate, was selected to target terpenoids and phenolic compounds, known to display interesting antioxidant activities [10]. Even if effective, the use of organic solvents brings numerous disadvantages, mostly related to the negative environmental impact of their production and disposal.

Following global trends, where the interest in “green” products and technologies is growing, this work presents a supercritical CO_2_ extraction methodology, a technique easily scalable and applicable at the industrial level, as the core strategy characterized by a low environmental impact: the use of organic solvents is reduced and carbon dioxide is used as the extraction fluid, ensuring a safe and selective extraction strategy, with the possibility to recycle the employed CO_2_ in industrial plants [19]. Since sc-CO_2_ displays a solvating power similar to *n*-hexane, the first sets of parameters, based on CO_2_ alone, were not able to provide a remarkable mass gain in terms of yield (only a maximum of 0.11% yield was achieved, see Figure 1). Samples were characterized by a waxy consistency and solubility in apolar solvent, such as *n*-hexane or dichloromethane.

Instead, the addition of ethanol as a cosolvent in a relatively low ratio was able to modify the supercritical fluid polarity, allowing to shift the window of extractables towards more polar molecules [20]. In fact, as shown in Figure 1, the cumulative yield displayed a net increment when ethanol was added during the supercritical extraction, enabling to reach a 1.1% yield, a value similar to the yield obtained during conventional extraction.

Fractions deriving from each static/dynamic cycle in supercritical CO_2_ were collected separately in order to study their differences in chemical profiles and antioxidant activity. In Figure 1, the samples SFE1 and SFE6, selected for further investigation, are highlighted. Sample SFE1 was selected, because it was the first extract obtained in the presence of ethanol as a cosolvent, whereas sample SFE6 was the second-last extract obtained with ethanol as a cosolvent before the extraction was stopped. The very last extract was excluded from further investigation, because it might contain some residuals collected in the vial during the vessel depressurization and, thus, could not be a valuable representative of the extraction during the last part of the experiment.

Thus, EtOAc, SFE1 and SFE6 samples were subjected to HPLC-ESI-MS analysis in order to identify their chemical compositions. The results indicated that the EtOAc extract contains more ursolic acid than SFE1 and SFE6, clearly suggesting that the supercritical fluid extraction, although modified by the small percentage of ethanol, is not very efficient in extracting and concentrating this triterpenoid. On the contrary, SFE1 contains more flavonoids than SFE6 and EtOAc. According to Table S1 and Figure 3, the main flavonols in all the extracts were glycosylated forms of quercetin and isorhamnetin, with also a pair of isorhamnetin 3-O acetyl hexosides.

The literature clearly suggests that flavonoids, along with triterpenoids from pears, possess some biological activities, such as the antioxidant one that has been measured only employing the DPPH, ABTS and FRAP assays, respectively [3,4]. Thus, in agreement with previous studies [3,4], the EtOAc, SFE1 and SFE6 samples are more efficient in the reduction of ABTS than DPPH radicals, respectively, whereas EtOAc displays a better FRAP activity compared to the other two extracts (Figure 4A–C).

These preliminary results prompted us to better investigate the antioxidant activity of the three extracts at the cellular level. In this context, human Caco-2 cells were used as a cellular model able to reproduce in vitro the intestinal system, because in the absorption of nutrients and in the immune response, the gastrointestinal tract plays a key role in the production of ROS, and several pieces of evidence highlight how the pathogenesis of various gastrointestinal tract diseases are, in part, due to oxidative stress [21]. Hence, H_2_O_2_ was used as a stimulus for inducing oxidative stress on Caco-2 cells, and the rescue properties of each extract were investigated, measuring their ability to restore the normal level of intracellular ROS, lipid peroxidation and NO levels via modulation of the iNOS pathway. Indeed, the results clearly indicated that all the extracts restored the intracellular H_2_O_2_-induced ROS towards the physiological levels, thus leading to a reduction of H_2_O_2_-induced lipid peroxidation. From the statistical analysis, EtOAc is more active in reducing H_2_O_2_-induced ROS than SFE1 and SFE6 (**, *p* < 0.01) and in decreasing the H_2_O_2_-induced lipid peroxidation than SFE6 (*, *p* < 0.5), whereas any statistical difference was observed compared to SFE1. A previous study demonstrated that pear extracts from Nanguo pear, Aksu Juju pear, Hongxiang pear and Luntai Juju pear reduced the ROS levels in HEK-293, suggesting an antioxidant activity at the cellular level [22].

As regards the modulation of the NO production levels, the evidence underlines that both SFE1 and SFE6 were more active in the reduction towards the physiological conditions of the NO levels than EtOAc (Figure 6A; *, *p* < 0.5), thus suggesting a minor role of ursolic acid, which was in a much greater amount in EtOAc. All of extracts were similarly able to modulate the iNOS pathway (Figure 6B).

## 5. Conclusions

The use of agri-food waste as alternative raw material for the extraction of bioactive compounds is becoming an attractive strategy to develop nutraceuticals and food supplements. However, the risk-assessment of biowaste derived products is still scarcely diffuse, but when available, it confirms the safety of these products [23]. Hence, in order to properly valorize our pear by-product extracts for a practical exploitation by the pharmaceutical/nutraceutical/food industry, dedicated experiments have to be assessed for confirming the in vivo efficacy and safety overall applying a multidisciplinary strategy, this study provides in vitro evidences regarding the antioxidant activity of Italian pear by-product derived extracts which might be exploited as ingredients for the development of new dietary supplements and/or functional foods. Of course, we are aware that this is the first step of this process and that doubtlessly, an in vivo study on animal models would be necessary for obtaining the proof of concept of the safety of the ingredients and the health-promoting activity of these extracts. Notwithstanding, we are also aware that this study provides a practical strategy for making the Mantuan PGI pear supply chains more sustainable and closer to the SDG of the Agenda 2030 and Zero Waste principles and concepts, respectively.

## Data Availability

Not applicable.

## Supplementary Materials

The following supporting information can be downloaded at: https://www.mdpi.com/article/10.3390/antiox12010144/s1: Table S1: Phenolic compounds identified in each extract. Figure S1: Some representative UV–Vis spectra of the flavonol detected in the extracts. Figure S2: Effect of pear-derived extracts on cell vitality.
