# Supplementary material for: Valorization of the Antioxidant Effect of Mantua PGI Pear By-Product Extracts: Preparation, Analysis and Biological Investigation
Source: Antioxidants (Basel). 2023 Jan 7;12(1):144. doi: 10.3390/antiox12010144 (PMC9854704; doi:10.3390/antiox12010144)
Supplement: Supplementary file 1 [file antioxidants-12-00144-s001.zip › antioxidants-2105668-supplementary.pdf]

Supplementary Materials

Valorization of the antioxidant effect of Mantua PGI pear by-product extracts: preparation, analysis and biological investigation

Carlotta Bollati[1], Stefania Marzorati[2], Lorenzo Cecchi[3], Martina Bartolomei[1], Jianqiang Li[1], Maria Bellumori[3], Lorenza d'Adduzio[1], Luisella Verotta[2], Laura Piazza[2], Anna Arnoldi[1], Nadia Mulinacci[3], Carmen Lammi[1*]

[1] Department of Pharmaceutical Sciences, University of Milan, 20133 Milan, Italy
[2] Department of Environmental Science and Policy, Università degli Studi di Milano, 20133 Milan, Italy
[3] Department of Neuroscience, Psychology, Drug and Child Health, Pharmaceutical and Nutraceutical Section, University of Florence, 50019 Florence, Italy
* Correspondence: carmen.lammi@unimi.it; Tel.: +39-02-50319372

Table S1. List of the flavonols identified in the pear extracts by the evaluation of their UV-Vis and mass spectra obtained in negative ionization mode according to Brahem et al., 2017 [1].

	Flavonols	Rt (min)	MS neg, fr. 200	γ_{max} (nm)
**1**	Quercetin 3-O glucoside or galactoside	21.0	463, 301	255, 350
**2**	Quercetin 3-O glucoside or galactoside	21.4	463, 301	255, 350
**3**	Isorhamnetin 3-O glucorhamnoside	22.9	623, 315	255, 354
**4**	Isorhamnetin 3-O glucorhamnoside	23.3	623, 315	255, 355
**5**	Isorhamnetin 3-O glucoside or galactoside	23.8	477, 315	255, 355
**6**	Isorhamnetin 3-O glucoside or galactoside	24.3	477, 315	252, 354
**7**	Isorhamnetin 3-O acetylglucoside or acetylgalactoside	25.3	519, 315	254, 354
**8**	Isorhamnetin 3-O acetylglucoside or acetylgalactoside	25.9	519, 315	254, 354

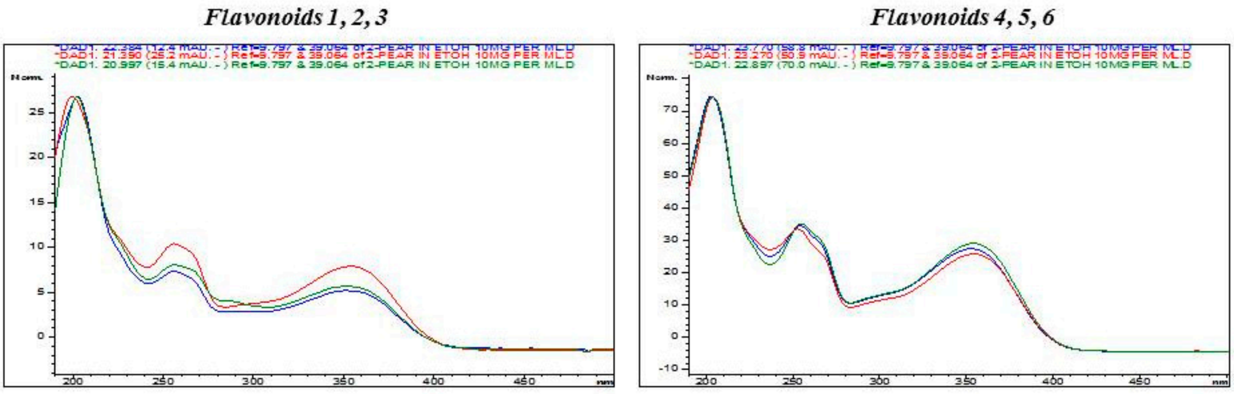

Figure S1. Some representative UV-Vis spectra of the flavonol detected in the extracts.

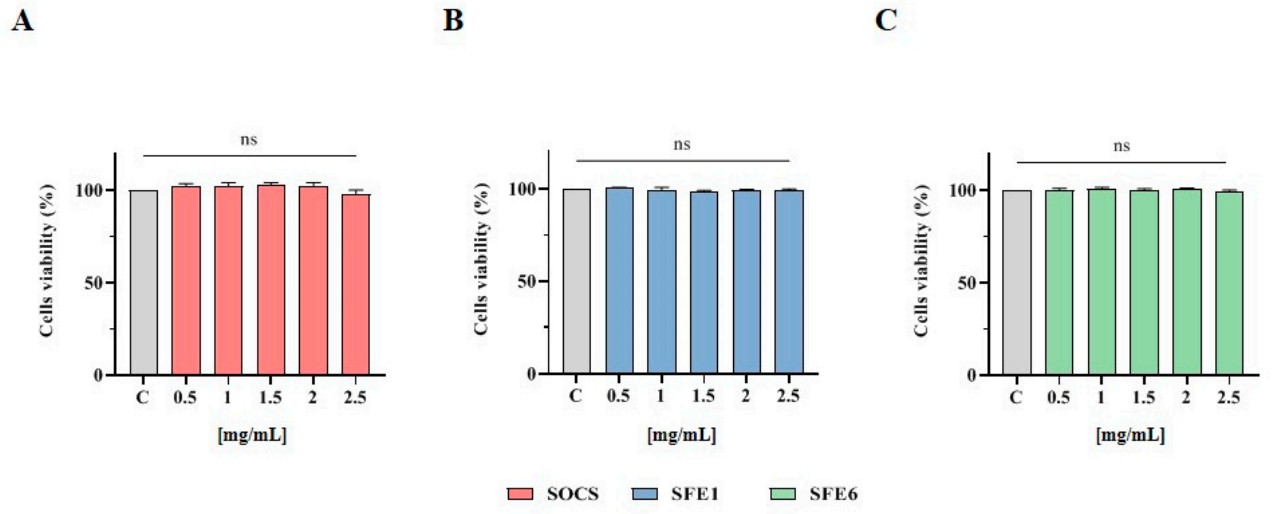

Figure S2: Bar graphs indicating the results of MTT cell viability assay of Caco-2 cells after SOCS, SFE1 and SFE6 extracts (0.5 - 2.5 mg/mL) treatments for 48 h. The data points represent the averages ± SEM of three experiments in triplicate. C: control (untreated) cells, ns: not significant.

2. Materials and Methods

2.1. Chemicals

Dulbecco's modified Eagle's medium (DMEM), L-glutamine, fetal bovine serum (FBS), phosphate buffered saline (PBS), penicillin/streptomycin, chemiluminescent reagent, and 24 or 96-well plates were purchased from Euroclone (Milan, Italy). MTT [3-(4,5-dimethylthiazol-2-yl)-2,5-diphenyltetrazolium bromide], DPPH (1,1-diphenyl-2-picrylhydrazyl), 2,2-azino-bis-(3-ethylbenzothiazoline-6-sulfonic acid (ABTS), TPTZ, Griess reagent, bovine serum albumin (BSA), RIPA buffer, the antibody against β-actin, fluorometric intracellular ROS kit and MDA assay kit were bought from Sigma-Aldrich (St. Louis, MO, USA). Phenylmethanesulfonyl fluoride (PMSF), Na-orthovanadate inhibitors, and the antibodies against rabbit Ig-horseradish peroxidase (HRP) and mouse Ig-HRP were purchased from Santa Cruz Biotechnology Inc. (Santa Cruz, CA, USA). The iNOS primary antibody came from Cell Signaling Technology (Danvers, MA, USA); the inhibitor cocktail Complete Midi from Roche (Basel, Swiss); Mini protean TGX pre-cast gel 7.5% and Mini nitrocellulose Transfer Packs from BioRad (Hercules, CA, USA). Ultrapure water was produced using a Milli-Q-system (Millipore SA, Molsheim, France). Acetonitrile with HPLC grade was from Panreac (Barcelona, Spain); formic acid was from Merck (Darmstadt, Germany). Standards of quercitrin (> 98.5%) and ursolic acid (> 98.0%) were from Extrasynthese (Genay, France).

2.2. Pear Extract Preparation

2.2.1. Starting Biomass

The pear waste biomass (*Pyrus communis L.* cultivar 'Abate Fétel', 3 kg) was provided by CREA (Consiglio per la Ricerca in Agricoltura e l'Analisi dell'Economia Agraria) after a sliding process to produce dried pear disks to be consumed as food. Prior to the drying process, the upper and lower part of each fresh pear, containing the skin and the core pulp with seeds and stalks, characterized by a non-compliant disk diameter, was discarded as a waste and stored at -20 °C in inert atmosphere. Pear waste were chopped and then lyophilized for 24 h using a Cinquepascal Srl (Trezzano Sul Naviglio, Italy) freeze dryer equipment. The lyophilized material was then ground using a knife mill (Fritsch, Pulverisette 11, Fritsch GmbH - Milling and Sizing, Idar-Oberstein, Germany) at 10,000 rpm for 20 s. To avoid powder heating during blending and the consequent degradation of thermally unstable species, liquid nitrogen was added. The pulverized biomass was then stored at -20 °C.

2.2.2. Extractions

Solvent-based Extraction

The lyophilized and pulverized biomass (19.4 g) was transferred in a 500 mL round flask and extracted with 150 mL of ethyl acetate, heating up to the boiling point with continuous stirring with a magnetic stirrer at reflux for 2.5 h. After cooling to room temperature, the suspension was filtered by means of a Buchner funnel to remove the solid particles. The extract (EtOAc) was then dried by a rotary evaporator (37 °C) and finally by a mechanical vacuum pump. The extraction was run in triplicate

Supercritical Fluid Extraction

Supercritical fluid extractions were performed using a pilot unit SFT110XW System supplied by Supercritical Fluid Technologies Inc. (Newark, DE, USA). It consisted of a 100 cm^3 stainless steel extractor inserted in an oven, a constant pressure piston pump (SFT-Nex10 SCF Pump) with a Peltier cooler, a Waters 515 HPLC pump for the co-solvent and a collection vial. The lyophilized and pulverized biomass (40.7 g) was loaded in the vessel for supercritical fluid extraction. The restrictor temperature was set at 75 °C. Sequentially different conditions [changing the pressure (p), temperature (T), and/or the amount of co-solvent] were applied, each one comprising an alternation of 15 min in static conditions (maceration in supercritical CO_2) followed by 30 min in dynamic conditions (flow rate CO_2 = 8.0 SCFH, standard cubic feet per hour):

- p=150 bar, T= 40 °C (d_{CO2} = 780.6 kg/m^3). The alternation of 3 static/dynamic cycle was enough to exhaust the extractables in these conditions, no evident mass gain was further achieved (SF1).
- p=300 bar, T= 40 °C (d_{CO2} = 909.3 kg/m^3). These conditions were not able to provide any extract mass gain.
- p=300 bar, T= 60 °C (d_{CO2} = 829.5 kg/m^3). These conditions were not able to provide any extract mass gain.
- p=300 bar, T= 80 °C (d_{CO2} = 746.1 kg/m^3). The alternation of 1 static/dynamic cycle was enough to exhaust the extractables in these conditions, no evident mass gain was further achieved.
- p=300 bar, T= 60 °C, co-solvent=ethanol (10% v_{EtOH} / $v_{sc\text{-}CO2}$). An alternation of 7 static/dynamic cycles was carried out (SF6).

Samples were dried by a rotary evaporator (37 °C) and finally by mechanical vacuum pump. Only the extracts deriving from the first (SFE1) and second-last (SFE6) cycles in the presence of ethanol were considered promising samples and were thus further investigated.

2.2.3. Waxes Removal

Before analysis, the three samples EtOAc, SFE1, and SFE6 were subjected to a procedure aimed at removing the waxes. Briefly, 2 mL of ethanol were added for every 100 mg of sample. The solution/suspension was warmed up to 40 °C for 3 min while stirring and then stored at −20 °C for 24 h. The solid-liquid mixture was then cold-filtered, the solvent was evaporated from the filtered solution by rotary evaporator and the residue was dried under vacuum pump for 4 h.

2.3. HPLC-DAD-MS Analysis of Pear Extracts

The dried extracts of the three samples were dissolved in ethanol to obtain a final concentration of 10 mg/mL each. The analyses were carried out using a 1260 Infinity II LC System coupled with both a Diode Array Detector and a Mass Spectrometry Detector (InfinityLab LC/MSD) with an API electrospray interface (all from Agilent Corporation, Santa Clara, CA, USA). The column was a Poroshell 120, EC-C18 (150 mm × 3.0 mm id, 2.7 μm, Agilent Technologies). The mobile phase was constituted by acetonitrile (A) and water at pH 3.2 by formic acid (B); the flow rate was 0.4 mL min^{-1}. A multistep linear gradient was applied: it started with A 5% at 0 min, from 5% to 40% in 40 min, a plateau at 40% until 45 min, then A 70% at 50 min, a plateau until 60 min, finally to A 100% at 65 min, and the last step was a plateau until 68 min; the system returned at A 5% in 2 min. The chromatograms were recorded at wavelengths set at 210, 240, 280, and 350 nm.

The mass spectra were acquired in negative ion mode applying the following conditions: range mass acquisition 100-1000 Dalton, gas temperature 350 °C, nitrogen flow rate 12 L/min, nebulizer pressure 35 psi, capillary voltage 3500 V, and fragmentation energy between 100 and 200 V.

The total content of flavonoids was evaluated at 350 nm by the calibration line of quercitrin at 350 nm (linearity range 0–0.49 μg, R^2 = 0.9999). Ursolic acid (a triterpenoid compound) was determined using a calibration curve at 220 nm (linearity range 0–12 μg; R^2 = 0.9999) of the pure standard.

2.4. DPPH (2,2-diphenyl-1-picrylhydrazyl radical scavenging) assay

1,1-Diphenyl-2-picrylhydrazyl radical (DPPH) assay was performed to determine the antioxidant activity by standard method [2] with a slight modification. Briefly, the DPPH solution (12.5 μM in methanol, 45 μL)

was added to 15 μL of EtOAc, SFE1 and SFE6 samples at different concentrations (0.1 – 5 mg/mL) in a 96-well half area plate. The reaction for scavenging DPPH radicals was performed in the dark at room temperature and the absorbance was measured at 520 nm after 30 min incubation.

2.5. TEAC assay

The TEAC assay is based on the reduction of the 2,2-azino-bis-(3-ethylbenzothiazoline-6-sulfonic acid (ABTS) radical induced by antioxidants [3]. The ABTS radical cation ($ABTS^{+\bullet}$ was prepared by mixing a 7 mM ABTS solution (Sigma-Aldrich, Milan, Italy) with 2.45 mM potassium persulfate (1:1) and stored for 16 h at room temperature and in dark. To prepare the ABTS reagent, the $ABTS^{+\bullet}$ was diluted in 5 mM phosphate buffer (pH 7.4) to obtain a stable absorbance of 0.700 (±0.02) at 730 nm. For the assay, 10 μL of EtOAc, SFE1 and SFE6 samples (at the final concentrations of 0.1, 0.5 and 1 mg/mL) were added to 140 μL of diluted the $ABTS^{+\bullet}$. The microplate was incubated for 30 min at 30 °C and the absorbance was read at 730 nm using a Synergy™ HT-multimode microplate reader (Biotek Instruments, Winooski, VT, USA)

2.6. FRAP assay

The FRAP assay evaluates the ability of a sample to reduce ferric ion (Fe^{3+}) into ferrous ion (Fe^{2+}) [4]. Thus, 10 μL of the sample (15×) were mixed with 140 μL of FRAP reagent. The FRAP reagent was prepared by mixing 1.3 mL of a 10 mM TPTZ (Sigma-Aldrich, Milan, Italy) solution in 40 mM HCl, 1.3 mL of 20 mM $FeCl_3 \times 6H_2O$ and 13 mL of 0.3 M acetate buffer (pH 3.6). The microplate was incubated for 30 min at 37 °C and the absorbance was read at 595 nm. The absorbance was recorded on a Synergy™ HT-multimode microplate reader.

2.7. Cell culture

Caco-2 cells, obtained from INSERM (Paris, France), were routinely sub-cultured at 50% density and maintained at 37 °C in a 90% air/10% CO_2 atmosphere in DMEM containing 25 mM of glucose, 3.7 g/L of $NaHCO_3$, 4 mM of stable L-glutamine, 1% nonessential amino acids, 100 U/L of penicillin, and 100 μg/L of streptomycin (complete medium), supplemented with 10% heat-inactivated fetal bovine serum (FBS; Hyclone Laboratories, Logan, UT, USA) following the procedure previously reported [5].

2.8 3-(4,5-Dimethylthiazol-2-yl)-2,5-Diphenyltetrazolium Bromide (MTT) Assay

The MTT experiments were conducted on Caco-2 cells, following the procedure previously reported [6]. Brefly, a total of 3 × 10^4 Caco-2 cells/well were seeded in 96-well plates and treated with EtOAc, SFE1 and SFE6 from 0.5 to 2.5 mg/mL, or vehicle, in complete growth media for 48 h at 37 °C under 5% CO_2 atmosphere. Subsequently, the treatment was aspirated and 100 μL/well of 3-(4,5-dimethylthiazol-2-yl)-2,5-diphenyltetrazolium bromide (MTT) filtered solution added. After 2 h of incubation at 37 °C under 5% CO_2 atmosphere, 0.5 mg/mL solution was aspirated and 100 μL/well of the lysis buffer (8 mM HCl + 0.5% NP-40 in DMSO) added. After 10 min of slow shaking, the absorbance at 575 nm was read on the Synergy H1 fluorescence plate reader (Biotek, Bad Friedrichshall, Germany).

2.9. Nitric Oxide Level Evaluation on Caco-2 Cells

Caco-2 cells (1.5 × 10^5/well) were seeded on 24-well plates. The next day, cells were treated for 24 h with EtOAc, SFE1 or SFE6 to reach the final concentrations of 2.0 mg/mL, and incubated at 37 °C under a 5% CO_2 atmosphere. After incubation, cells were treated with H_2O_2 (1.0 mM) or vehicle for 1 h, then the cell culture media were collected and centrifuged at 13,000 g for 15 min to remove insoluble material. NO determination was carried out by Griess test. Briefly, 1.0 g of Griess reagent powder were dissolved in 25.0 mL of distilled H_2O and 50.0 μL of the solution were incubated with 50.0 μL of the culture supernatants for 15 min at RT in the dark. The absorbance was measured at 540 nm using the Synergy H1 fluorescent plate reader from Biotek.

2.10. Fluorometric Intracellular ROS Assay

For cells preparation, 3 × 10^4 Caco-2 cells/well were seeded on a black 96-well plate overnight in growth medium. The day after, the medium was removed and replaced with 50 μL/well of the Master Reaction Mix and the cells were incubated at 5% CO_2, 37 °C for 1 h in the dark. Then, cells were treated with 5 μL of EtOAc, SFE1, SFE6 (to reach the final concentrations of 2 mg/mL) and incubated at 37 °C for 2 h in the dark. To induce

ROS, cells were treated with 5 μL of H_2O_2 at a final concentration of 1.0 mM for 30 min a 37 °C in the dark and fluorescence signals (ex./em. 490/525 nm) were recorded using a Synergy H1 microplate reader.

2.11. Lipid peroxidation (MDA) assay

Caco-2 cells (2.5 x 10^5 cells/well) were seeded in a 24 well plate and, the following day, they were treated with 2.0 mg/mL of EtOAc, SFE1, or SFE6 for 2 h at 37 °C under 5% CO_2 atmosphere. After incubation, cells were incubated with 1 mM H_2O_2 or vehicle, for 30 min, then collected and homogenized in 150 μL ice-cold MDA lysis buffer containing 1.5 μL of BHT (100x). Samples were centrifuged at 13,000 × g for 10 min, then they were filtered through a 0.2 μm filter to remove insoluble material. To form the MDA-TBA adduct, 300 μL of the TBA solution were added into each vial containing samples and incubated at 95 °C for 60 min, then cooled to RT for 10 min in an ice bath. For analysis, 100 μL of each reaction mixture were pipetted into a 96 well plate and the absorbance was measured at 532 nm using the Synergy H1 fluorescent plate reader from Biotek.

2.12. Western blot analysis

1.5 x 10^5 Caco-2 cells/well (24-well plate) were treated with 2 mg/mL of EtOAc, SFE1 and SFE6 for 24 h. After incubation, cells were treated with H_2O_2 (1.0 mM) or vehicle for 1 h, then the cell culture media were collected in an ice-cold microcentrifuge tube and processed for the Griess assay. Meanwhile the cells were scraped in 30 μL ice-cold lysis buffer [RIPA buffer + inhibitor cocktail + 1:100 PMSF + 1:100 Na-orthovanadate] and transferred in an ice-cold microcentrifuge tube. After centrifugation at 16,060 g for 15 min at 4 °C, the supernatant was recovered and transferred in a new ice-cold tube. Total proteins were quantified by the Bradford method and 50 μg of total proteins loaded on a pre-cast 7.5% sodium dodecyl sulfate - polyacrylamide (SDS-PAGE) gel at 130 V for 45 min. Subsequently, the gel was pre-equilibrated with 0.04% SDS in H_2O for 15 min at room temperature (RT) and transferred to a nitrocellulose membrane (Mini nitrocellulose Transfer Packs), using a Trans-blot Turbo at 1.3 A, 25 V for 7 min. Target proteins, on milk or BSA blocked membrane, were detected by primary antibodies as follows: anti-iNOS and anti-β-actin. Secondary antibodies conjugated with HRP and a chemiluminescent reagent were used to visualize target proteins and their signal was quantified using the Image Lab Software (Biorad). The internal control β-actin was used to normalize loading variations.

2.13. Statical Analysis

All results were expressed as the mean ± standard deviation (s.d.), where p-values < 0.05 were considered to be significant. Statistical analyses were performed by one- and two-way ANOVA followed by Tukey's post-test, respectively (Graphpad Prism 9, GraphPad Software, La Jolla, CA, USA).

Referecnces

1. Brahem, M.; Renard, C.M.G.C.; Eder, S.; Loonis, M.; Ouni, R.; Mars, M.; Le Bourvellec, C. Characterization and Quantification of Fruit Phenolic Compounds of European and Tunisian Pear Cultivars. *Food Res. Int.* **2017**, doi:10.1016/j.foodres.2017.03.002.
2. Blois, M.S. Antioxidant Determinations by the Use of a Stable Free Radical [10]. *Nature* 1958.
3. Miller, N.J.; Rice-Evans, C.; Davies, M.J.; Gopinathan, V.; Milner, A. A Novel Method for Measuring Antioxidant Capacity and Its Application to Monitoring the Antioxidant Status in Premature Neonates. *Clin. Sci.* **1993**, doi:10.1042/cs0840407.
4. Benzie, I.F.F.; Strain, J.J. The Ferric Reducing Ability of Plasma (FRAP) as a Measure of "Antioxidant Power": The FRAP Assay. *Anal. Biochem.* **1996**, doi:10.1006/abio.1996.0292.
5. Lammi, C.; Bollati, C.; Gelain, F.; Arnoldi, A.; Pugliese, R. Enhancement of the Stability and Anti-DPPIV Activity of Hempseed Hydrolysates through Self-Assembling Peptide-Based Hydrogels. *Front. Chem.* **2019**, doi:10.3389/fchem.2018.00670.
6. Li, Y.; Aiello, G.; Bollati, C.; Bartolomei, M.; Arnoldi, A.; Lammi, C. Phycobiliproteins from Arthrospira Platensis (Spirulina): A New Source of Peptides with Dipeptidyl Peptidase-IV Inhibitory Activity. *Nutrients* **2020**, doi:10.3390/nu12030794.
